# Does Instruction of Oral Health Behavior for Workers Improve Work Performance?—Quasi-Randomized Trial

**DOI:** 10.3390/ijerph15122630

**Published:** 2018-11-24

**Authors:** Naoki Toyama, Ayano Taniguchi-Tabata, Nanami Sawada, Yoshio Sugiura, Daiki Fukuhara, Yoko Uchida, Hisataka Miyai, Aya Yokoi, Shinsuke Mizutani, Daisuke Ekuni, Manabu Morita

**Affiliations:** 1 Department of Preventive Dentistry, Okayama University Graduate School of Medicine, Dentistry and Pharmaceutical Sciences, Okayama University, Okayama 700-8558, Japan; pu171qxi@s.okayama-u.ac.jp (N.T.); de422027@s.okayama-u.ac.jp (N.S.); de421022@s.okayama-u.ac.jp (Y.S.); de20041@s.okayama-u.ac.jp (D.F.); de20006@s.okayama-u.ac.jp (Y.U.); pqpp0n70@s.okayama-u.ac.jp (H.M.); yokoi-a1@cc.okayama-u.ac.jp (A.Y.); dekuni7@md.okayama-u.ac.jp (D.E.); mmorita@md.okayama-u.ac.jp (M.M.); 2 Department of Preventive Dentistry, Okayama University Hospital, Okayama 700-8558, Japan; 3 Section of Geriatric Dentistry and Perioperative Medicine in Dentistry, Division of Maxillofacial Diagnostic and Surgical Sciences, Faculty of Dental Science, Kyushu University, Fukuoka 812-8582, Japan; mizutani@dent.kyushu-u.ac.jp; 4 OBT Research Center, Faculty of Dental Science, Kyushu University, Fukuoka 812-8582, Japan

**Keywords:** work performance, oral health, intervention study, behavioral modification

## Abstract

Oral disease can cause economic loss due to impaired work performance. Therefore, improvement of oral health status and prevention of oral disease is essential among workers. The purpose of this study was to investigate whether oral health-related behavioral modification intervention influences work performance or improves oral health behavior and oral health status among Japanese workers. We quasi-randomly separated participants into the intervention group or the control group at baseline. The intervention group received intensive oral health instruction at baseline and a self-assessment every three months. Both groups received oral examinations and answered the self-questionnaire at baseline and at one-year follow-up. At follow-up, the prevalence of subjects who use fluoride toothpastes and interdental brushes/dental floss were significantly higher in the intervention group than in the control group. Three variables (tooth brushing in workplace, using fluoride toothpaste, and experience of receiving tooth brushing instruction in a dental clinic) showed significant improvement only in the intervention group. On the other hand, work performance and oral status did not significantly change in either group. Our intensive oral health-related behavioral modification intervention improved oral health behavior, but neither work performance nor oral status, among Japanese workers.

## 1. Introduction

Health impairment influences work performance due to pain, absence for treatment and physical disability [1,2,3,4]. The World Health Organization states that protecting workers’ health is important to household income, productivity, and economic development, and work-related health problems result in an economic loss of 4–6% of gross domestic product (GDP) for most countries [5]. 

Several chronic diseases, including oral diseases, were reported to cause economic loss due to impaired work performance [6]. In Japan, 34.8% of workers had problems with work due to oral diseases and impaired work performance [7]. Another study reported that oral diseases indirectly impose an economic burden, costing US$144 billion in terms of productivity losses due to absenteeism from work [8]. Thus, prevention and control of oral diseases is important for workers to avoid impaired work performance and subsequent economic loss.

Improving individual oral health behavior is effective for preventing oral diseases. Dentists or dental hygienists perform behavioral modification for improvement of patient oral health behavior [9,10,11]. Adopting methods for behavioral modification, such as “prompt self‒monitoring of behavior”, “prompt intention formation”, “prompt specific goal setting”, “provide feedback on performance”, and “prompt review of behavioral goals”, are effective [12,13]. However, there have been few studies investigating the effects of intervention for behavioral modification on work performance. 

We hypothesize that oral health-related behavioral modification intervention will improve work performance by improving oral health behavior and oral health status. This study aims to investigate whether oral health-related behavioral modification intervention influences work performance or improves oral health behavior and oral health status among Japanese workers.

## 2. Materials and Methods 

### 2.1. Study Population

We estimated the sample size using G*Power and calculated minimum sample sizes for a chi-squared test. We set the effect size at 0.3, alpha at 0.05, and power (1 − β) at 0.80 [14]. The minimum sample size was 108 (chi-squared test). Assuming an attrition rate of 30% [15,16], the planned sample size was therefore a minimum of 308 participants (154 in each group).

Among central or branch offices in Okayama in Japan, we recruited companies that have never received oral examination in work places and agreed to participate in the study. A total of 14 companies in Okayama, Hiroshima, Osaka, and Kyoto cities in Japan agreed to participate in this study. Inclusion criteria for participant recruitment were to complete oral examinations and questionnaires, while exclusion criteria were participants who did not agree to participate. We enrolled 611 workers from April to December 2015 and performed re-examination from April to December 2016. 

This study was an assessor-blinded, quasi-randomized trial (alternate allocation). All participants first received an oral examination and answered self-administered questionnaires, and were then divided into two groups in the order in which they came at baseline (2015). After alternate allocation (ratio; 1:1), participants were assigned to the intervention group or the control group. After oral examination, the intervention group received instructions for oral health-related behavioral modification. They were involved in further intervention by the mailing method, which was performed every three months. The control group received only oral examinations. After one year (follow-up) (2016), the two groups received re-examination and answered self-questionnaires. 

All study protocols were approved by the Ethics Committees of Okayama University Graduate School of Medicine, Dentistry and Pharmaceutical Sciences and Okayama University Hospital (no. 1507-001). Written informed consent was obtained from all targeted participants. Moreover, this study was registered at the University Hospital Medical Information Network (no. 000023011) before commencing.

### 2.2. Oral Examination

At baseline and follow-up, six dentists (M.M., T.I., H.M., A.T.-T., A.Y., D.F.) who did not know the allocation performed oral examinations (single blind). The dentists assessed oral health status based on community periodontal index (CPI) [17], debris index-simplified (DI-S) [18] and bleeding on probing (BOP) using a CPI probe (YDM, Tokyo, Japan). CPI, DI-S, and BOP were measured for 10 representative teeth (maxilla: right first and second molar, right central incisor, left first and second molar; mandible: right first and second molar, left central incisor, left first and second molar). CPI scores were binarized; 0–2 vs. 3, 4. DI-S was evaluated in 4 grades (0–3). BOP was expressed as percentage (%BOP). In addition, the number of present teeth, decayed teeth, and filling teeth were recorded [17]. For assessment, all dentists received training and calibration. Data of CPI score (≤2/>2) were analyzed using a non-parametric kappa test. The kappa coefficients for intra- and inter-examiner reliability were 1.0 and 0.83, respectively.

### 2.3. Self-Questionnaire

Before oral examination, participants answered self-questionnaires on sex, age, job category [19], work pattern (daytime/daytime and nighttime/flextime), and 10 questions about oral health [20], as presented below:

(1) Do you have a family dental doctor? (Yes/No)
(2) Does your work disturb you going to dental clinic? (Yes/No)
(3) Do you brush your teeth in your workplace? (Always/Sometimes/No)
(4) Do you eat snack food between meals? (Always/Sometimes/No)
(5) Do you smoke tobacco? (Current smoking/Past smoking/Never)
(6) Do you brush your teeth before going to bed? (Always/Sometimes/No)
(7) Do you use fluoride toothpaste? (Yes/No/I don’t know)
(8) Do you use interdental brushes/dental floss? (Always/Sometimes/No)
(9) Have you received tooth brushing instruction at a dental clinic? (Yes/No)
(10) Have you received oral examination in the past year at a dental clinic? (Yes/No)

Furthermore, to assess whether oral status influences work performance, we asked “Have you had any problems with work performance because of oral diseases?” [7]. The answer was given in a “yes/no” format. If the answer was “yes”, work performance was assessed as impaired. 

### 2.4. Intervention

The intervention group received individualized instruction for five minutes. During the study briefing, the participants set three goals for oral health behavioral modification to improve individual oral status and received advice on achieving the goals using a leaflet and a dental model. The instructors were dental hygienists or dentists who did not perform oral examinations. Moreover, we performed self-assessment questionnaires three times per year by mail (mailing method). In the mailing method, the intervention group evaluated the level of achievement of the goals, which were suggested at baseline intervention and reconsidered the direction. If the goals were achieved, new goals were established by participants.

### 2.5. Statistical Analysis

SPSS version 20 software (IBM, Tokyo, Japan) was used for statistical analyses. Values of *p* < 0.05 were considered to indicate significant associations. Chi-squared tests or non-paired *t*-tests were used to assess whether there were significant differences between the intervention group and the control group at both baseline and follow-up. McNemar test, McNemar-Bowker tests or paired *t*-tests were used to assess whether there were significant changes between baseline and follow-up.

## 3. Results

**Figure 1:**
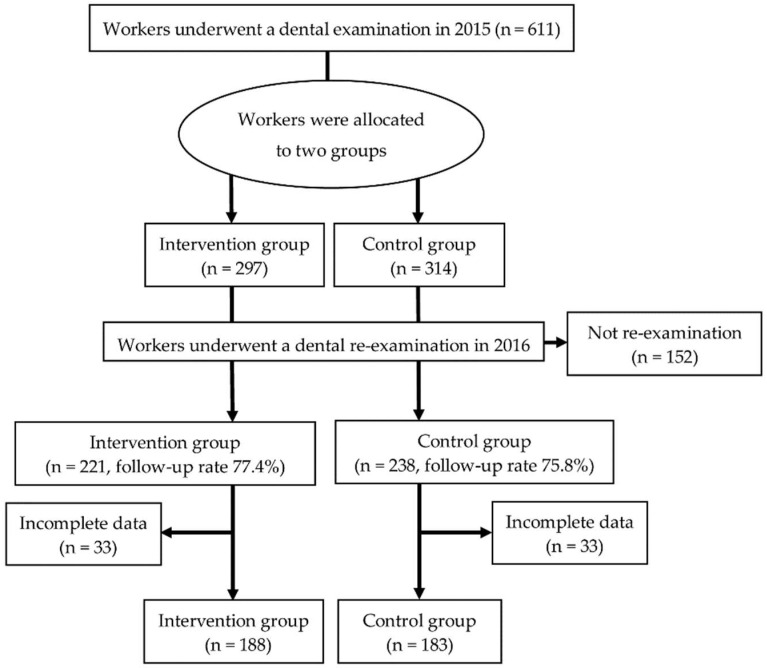
Flow chart showing the protocols for selecting analyzed workers from among those who agreed to participate in this study.

Figure 1 shows the flow chart for study participants. All participants agreed to participate in this study. As the participants who did not undergo re-examination or provided incomplete data were excluded, 371 workers out of 611 workers were included in the analysis (final follow-up rate; 60.7%).

Table 1 shows the distribution of participants’ characteristics at baseline. Data were not significantly different between the two groups at baseline (*p* ≥ 0.05, chi-squared tests, data not shown). The most common job category was professional and technical workers (36.9%). Daytime workers accounted for 85.4% of participants. 

In Table 2, we show a comparison of clinical variables between the two groups at baseline and at follow-up. All variables related to oral health status did not significantly differ between the two groups at baseline and follow-up (*p* ≥ 0.05, non-paired *t*-tests or chi-squared tests, data not shown).

The distribution of self-questionnaire answers between the intervention group and the control group is shown in Table 3. At baseline, there were no significant differences between the two groups (*p* ≥ 0.05, chi-squared tests, data not shown). After intervention, the frequency of fluoride toothpaste and interdental brushes/dental floss use was higher in the intervention group than in the control group (*p* < 0.05). There were no significant differences in work performance.

Changes in measured variables from baseline to follow-up in each group were also compared (Table 4). Use of interdental brushes/dental floss and dental examinations in the past year improved significantly in both groups. On the other hand, three variables (tooth brushing in workplace, using fluoride toothpastes, and experience of receiving tooth brushing instruction) showed significant improvement only in the intervention group. Work performance and oral status did not change significantly.

Oral health behavioral interventions are not invasive. Therefore, there were no study-related serious adverse events in this study. Furthermore, outcomes did not change after the trial commenced.

## 4. Discussion

To the best of our knowledge, this was the first study to assess changes in work performance after oral health-related behavioral modification intervention. The study design was reliable as examinations were performed blinded, participants were quasi-randomly (alternate allocation) separated into either an intervention group or a control group, and the sample size was sufficiently large. Unfortunately, this intervention did not improve work performance, and there are several reasons for this. In a previous study [21], it was reported that work performance is mainly influenced by pain from oral diseases. In this study, there was a significant association between work performance and oral pain (baseline, *p* = 0.002; follow-up, *p* = 0.019; chi-squared tests; data not shown). However, there was no significant difference in the decrease in oral pain between the intervention and control groups (*p* ≥ 0.05). A previous study showed that a combination of professional oral hygiene treatment and oral hygiene instructions contributed to a decrease in gingival-related pain [22]. Thus, in the future, we should investigate whether a combination of professional oral hygiene treatment and oral health instruction improves work performance.

Oral health-related behavioral modification intervention improved oral health behavior but not oral health status. A systematic review showed that oral hygiene instruction had short-term and long-term effects [10]. The short-term effects were improving knowledge, attitudes, self-efficacy, oral health behavior, and theory constructs. The long-term effects included improving the number of decayed teeth, plaque score, BOP, and gingival condition [10]. The results of this study may be included in the short-term effects. Menegaz et al. suggested that a follow-up time of less than one year led to a lack of efficacy for educational intervention [23]. In addition, Oshikohji et al. reported that workers who had more participation time for oral examination and oral health instruction had better periodontal condition than those with less time [24]. If the duration of this study and/or the frequency of instruction was increased, oral health status might improve. 

The intervention in this study was advantageous as it included some of the known factors that lead to behavioral modification. We explained why the workers should change their behavior (prompt intention formation), let the workers set goals independently (prompt specific goal setting), and checked their improvement and prompted them to reconsider their goals (prompt self‒monitoring of behavior and prompt review of behavioral goals) [12,13]. Goals to improve oral status were also set based on individual situations in this study. These concepts were supported by a previous study [25]. Finally, the intervention time was short (5 min), a factor which may be effective in workplaces to improve oral health behavior.

There were 17 participants who had problems with work because of tooth or gum disease (4.6% of participants) at baseline. These conditions agree with the prevalence of poor work performance caused by oral pain in previous studies, which ranged between 1.0–7.6% [25,26,27,28]. The percentage in this study was within this range. However, the job sector of participants in this study was skewed. The percentage of workers who belonged to the tertiary industry sector was high (83%), and there were no workers from the primary industry sector. Therefore, we should exercise caution when applying our results more generally.

There were some limitations with regard to the interpretation of these results. First, although most of the participants visited a dental clinic during the study period, the type of dental health instruction they received was not confirmed. The intensity of instruction may have affected the results. Second, the follow up rate was not high (approximately 60.7%). As >20% loss would pose a serious threat [29], the high percentage of loss to follow-up may have affected our results. In the intervention group, the ratios of work performance, oral status, and oral health behavior were not significantly different between the analyzed and non-analyzed workers (188 vs. 85 workers, chi-squared test and non-paired *t*-test, *p* > 0.05). However, in the control group, the percentage of those using interdental brushes/dental floss was significantly different (183 vs. 90 workers, chi-squared test, *p* = 0.034). In the control group, use of interdental brushes or dental floss might have been improved because more workers who did not use these were not analyzed. Other limitations include the short-term scale of the study period and the fact that this was not a randomized trial. 

## 5. Conclusions

In conclusion, oral health-related behavioral modification intervention improved oral health behavior, but not work performance in Japanese workers.

## Figures and Tables

**Table 1:** Characteristics of participants at baseline.

Variables	Intervention (*n* = 188)	Control (*n* = 183)
Sex		
Male	149 (79.3) ^1^	148 (80.9)
Female	39 (20.7)	35 (19.1)
Age (y)	40.7 ± 11.9 ^2^	41.4 ± 11.9
Job category		
Administrative and managerial workers	16 (8.5)	25 (13.7)
Professional and technical workers	67 (35.6)	70 (38.3)
Clerical workers	38 (20.2)	23 (12.6)
Sales workers	20 (10.6)	15 (8.2)
Service workers	3 (1.6)	4 (2.2)
Security workers	9 (4.8)	7 (3.8)
Manufacturing process workers	28 (14.9)	36 (19.7)
Transport and machine operation workers	7 (3.7)	3 (1.6)
Work schedule		
Daytime work	160 (85.1)	157 (85.8)
Daytime and nighttime work	8 (4.3)	6 (3.3)
Flextime work	20 (10.6)	20 (10.9)

^1^*n* (%); ^2^ Mean ± standard deviation.

**Table 2:** Comparison of clinical variables between the intervention group and the control group at follow-up.

Variables		Baseline (2015)		Follow-up (2016)		*p*-Value
		Intervention (*n* = 188)	Control (*n* = 183)	Intervention (*n* = 188)	Control (*n* = 183)	
DI-S ^1^		0.36 ± 0.34 ^4^	0.38 ± 0.33	0.35 ± 0.36	0.34 ± 0.37	0.784 ^6^
%BOP ^2^		39.0 ± 30.5	40.5 ± 28.4	36.3 ± 27.3	37.4 ± 26.3	0.674
Present teeth		28.3 ± 2.4	28.5 ± 1.8	28.3 ± 2.4	28.6 ± 1.9	0.246
Decayed teeth		0.69 ± 1.58	0.77 ± 2.06	0.64 ± 1.60	0.60 ± 1.56	0.796
Filling teeth		8.67 ± 6.08	9.06 ± 5.73	8.66 ± 6.13	9.20 ± 5.64	0.376
CPI ^3^	≤2	113 (60.1) ^5^	110 (60.1)	121 (64.4)	116 (63.4)	0.845 ^7^

^1^ Debris index-simplified; ^2^ Percentage of bleeding on probing; ^3^ Community periodontal index; ^4^ Mean ± standard deviation; ^5^ *n* (%); ^6^ Non-paired *t*-test at follow-up; ^7^ Chi-squared test at follow-up.

**Table 3:** Comparison of qualitative variables between the intervention group and the control group at follow-up.

Vriables	Baseline (2015)		Follow-up (2016)		*p*-Value ^1^
	Intervention (*n* = 188)	Control (*n* = 183)	Intervention (*n* = 188)	Control (*n* = 183)	
*Have you had any problems with work performance because of oral diseases?*					
Yes	9 (4.8) ^2^	12 (6.6)	7 (3.7)	7 (3.8)	0.959
No	179 (95.2)	171 (93.4)	181 (96.3)	176 (96.2)	
*Do you have a family dental doctor?*					
Yes	118 (62.8)	116 (63.4)	126 (67.0)	122 (66.7)	0.942
No	70 (37.2)	67 (36.6)	62 (33.0)	61 (33.3)	
*Does your work disturb you going to dental clinic?*					
Yes	91 (48.4)	76 (41.5)	83 (44.1)	71 (38.8)	0.296
No	97 (51.6)	107 (58.5)	105 (55.9)	112 (61.2)	
*Do you brush your teeth in your workplace?*					
Always	56 (29.8)	46 (25.1)	51 (27.1)	50 (27.3)	0.07
Sometimes	38 (20.2)	43 (23.5)	62 (33.0)	42 (23.0)	
No	94 (50.0)	94 (51.4)	75 (39.9)	91 (49.7)	
*Do you eat snack food between meals?*					
Always	43 (22.9)	45 (24.6)	43 (22.9)	52 (28.4)	0.344
Sometimes	115 (61.2)	111 (60.7)	114 (60.6)	108 (59.0)	
No	30 (16.0)	27 (14.8)	31 (16.5)	23 (12.6)	
*Do you smoke tobacco?*					
Current smoking	43 (22.9)	44 (24.0)	45 (23.9)	43 (23.5)	0.994
Past smoking	33 (17.6)	29 (15.8)	31 (16.5)	30 (16.4)	
Never	112 (59.6)	110 (60.1)	112 (59.6)	110 (60.1)	
*Do you brush your teeth before going to bed?*					
Always	152 (80.9)	137 (74.9)	160 (85.1)	140 (76.5)	0.075
Sometimes	25 (13.3)	28 (15.3)	21 (11.2)	28 (15.3)	
No	11 (5.9)	18 (9.8)	7 (3.7)	15 (8.2)	
*Do you use fluoride toothpaste?*					
Yes	95 (50.5)	82 (44.8)	124 (66.0)	96 (52.5)	0.029
No	36 (19.1)	46 (25.1)	34 (18.1)	44 (24.0)	
I don’t know	57 (30.3)	55 (30.1)	30 (16.0)	43 (23.5)	
*Do you use interdental brushes/dental floss?*					
Always	26 (13.8)	28 (15.3)	43 (22.9)	32 (17.5)	0.021
Sometimes	74 (39.4)	70 (38.3)	88 (46.8)	70 (38.3)	
No	88 (46.8)	85 (46.4)	57 (30.3)	81 (44.3)	
*Have you received tooth brushing instruction at a dental clinic?*					
Yes	129 (68.6)	129 (70.5)	146 (77.7)	135 (73.8)	0.382
No	59 (31.4)	54 (29.5)	42 (22.3)	48 (26.2)	
*Have you received an oral examination in the past year at a dental clinic?*					
Yes	72 (38.3)	65 (35.5)	89 (47.3)	87 (47.5)	0.969
No	116 (61.7)	118 (64.5)	99 (52.7)	96 (52.5)	

^1^ Chi-squared test on differences between intervention and control groups at follow-up; ^2^ *n* (%).

**Table 4:** Changes in variables in intervention and control groups.

Variables	Intervention (*n* = 188)			Control (*n* = 183)		
	Baseline	Follow-up	*p*-Value ^6^	Baseline	Follow-up	*p*-Value ^6^
Continuous variables						
DI-S ^1^	0.35 ± 0.36 ^4^	0.36 ± 0.34	0.913	0.38 ± 0.33	0.34 ± 0.37	0.165
%BOP ^2^	39.0 ± 30.5	36.3 ± 27.3	0.290	40.5 ± 28.4	37.4 ± 26.3	0.179
Present teeth	28.3 ± 2.36	28.3 ± 2.37	0.381	28.5 ± 1.78	28.6 ± 1.90	0.414
Decayed teeth	0.69 ± 1.58	0.64 ± 1.60	0.515	0.77 ± 2.06	0.60 ± 1.56	0.062
Filling teeth	8.67 ± 6.08	8.66 ± 6.14	0.969	9.06 ± 5.73	9.21 ± 5.64	0.337
	**Improved**	**Worsened**	***p*-Value ^7^**	**Improved**	**Worsened**	***p*-Value ^7^**
Categorical variables						
CPI ^3^	33 (17.6) ^5^	25 (13.3)	0.358	32 (17.5)	26 (14.2)	0.512
*Have you had any problems with work performance because of oral diseases?*						
	9 (4.8)	7 (3.7)	0.804	10 (5.5)	5 (2.7)	0.302
*Do you have a family dental doctor?*						
	13 (6.9)	5 (2.7)	0.096	18 (9.8)	12 (6.6)	0.362
*Does your work disturb you going to dental clinic?*						
	26 (13.8)	18 (9.6)	0.291	25 (13.7)	20 (10.9)	0.551
*Do you brush your teeth in your workplace?*						
	30 (16.0)	16 (8.5)	0.003	22 (12.1)	19 (10.4)	0.256
*Do you eat snack food between meals?*						
	27 (14.4)	26 (13.8)	0.997	18 (9.8)	28 (15.3)	0.403
*Do you smoke tobacco?*						
	4 (2.1)	7 (3.7)	0.392	8 (4.3)	7 (3.8)	0.978
*Do you brush your teeth before going to bed?*						
	13 (6.9)	4 (2.1)	0.132	13 (7.1)	10 (5.4)	0.733
*Do you use fluoride toothpaste?*						
	53 (28.2)	7 (8.0)	<0.001	35 (19.1)	22 (12.0)	0.076
*Do you use interdental brushes/dental floss?*						
	50 (26.6)	7 (3.7)	<0.001	26 (14.2)	15 (8.2)	0.049
*Have you received tooth brushing instruction at a dental clinic?*						
	24 (12.8)	7 (3.7)	0.003	16 (8.7)	10 (5.5)	0.327
*Have you received an oral examination in the past year at a dental clinic?*						
	28 (14.9)	11 (5.9)	0.009	32 (17.5)	10 (5.5)	0.001

^1^ Debris index-simplified; ^2^ Percentage of bleeding on probing; ^3^ Community periodontal index; ^4^ Mean ± standard deviation; ^5^ n (%); ^6^ Paired *t*-test; ^7^ McNemar test or McNemar-Bowker test.
